# Efficacy of Alveolar Ridge Preservation after Maxillary Molar Extraction in Reducing Crestal Bone Resorption and Sinus Pneumatization: A Multicenter Prospective Case-Control Study

**DOI:** 10.1155/2018/9352130

**Published:** 2018-11-04

**Authors:** Teresa Lombardi, Fabio Bernardello, Federico Berton, Davide Porrelli, Antonio Rapani, Alvise Camurri Piloni, Luca Fiorillo, Roberto Di Lenarda, Claudio Stacchi

**Affiliations:** ^1^Private Practice, Cassano allo Ionio (CS), Italy; ^2^Private Practice, Terranegra di Legnago (VR), Italy; ^3^Department of Medical, Surgical and Health Sciences, University of Trieste, Italy; ^4^Department of Biomedical and Dental Sciences, Morphological and Functional Images, School of Dentistry University of Messina, Messina, Italy

## Abstract

**Aim:**

To evaluate, with three-dimensional analysis, the effectiveness of alveolar ridge preservation (ARP) after maxillary molar extraction in reducing alveolar bone resorption and maxillary sinus pneumatization when compared to unassisted socket healing.

**Methods:**

Patients were included in the study following inclusion criteria and underwent minimally traumatic maxillary molar extraction followed by ARP using synthetic nanohydroxyapatite (Fisiograft Bone, Ghimas, Italy) (test group) or unassisted socket healing (control group). Cone-beam computerized tomographies (CBCT) were performed immediately after tooth extraction (T0) and 6 months postoperatively (T1). CBCTs were superimposed by using a specific software (Amira, Thermo Fisher Scientific, USA) and the following items were analyzed in both groups: (i) postextractive maxillary sinus floor expansion in coronal direction and (ii) postextractive alveolar bone dimensional changes (both vertical and horizontal). All data were tested for normality and equality of variance and subsequently analyzed by independent samples T-test and Mann–Whitney test.

**Results:**

Thirty patients were treated by three centers and twenty-six (test n=13; control n=13) were included in the final analysis. Mean sinus pneumatization at T1 was 0.69±0.48 mm in the test group and 1.04±0.67 mm in the control group (p=0.15). Mean vertical reduction of the alveolar bone at T1 was 1.62±0.49 mm in the test group and 2.01±0.84 mm in the control group (p=0.08). Mean horizontal resorption of crestal bone at T1 was 2.73±1.68 mm in test group and 3.63±2.24 mm in control group (p=0.24).

**Conclusions:**

It could be suggested that ARP performed after maxillary molar extraction may reduce the entity of sinus pneumatization and alveolar bone resorption, compared to unassisted socket healing. This technique could decrease the necessity of advanced regenerative procedures prior to dental implant placement in posterior maxilla.

## 1. Introduction

After dental extraction, the alveolar bone undergoes a remodeling process resulting in horizontal and vertical reduction of crestal dimensions [1, 2]. Ridge resorption may lead to inadequate bone volume for dental implants insertion and create both functional and esthetic issues during prosthetic rehabilitation [3]: in the posterior maxilla, in particular, a substantial percentage of edentulous patients may need bone augmentation procedures to allow a proper implant placement and reach satisfactory results [4]. Many surgical solutions are currently available to regenerate an adequate amount of bone in the atrophic crests, including lateral and transcrestal sinus floor elevation, guided bone regeneration, and block grafting [5–10]. However, all of these options are associated with significant rate of complications, increased morbidity, high costs, and prolonged time of therapy [11–13]. In the attempt to reduce the need for advanced surgical procedures and to simplify the treatment plan, specific surgical techniques were developed to reduce postextractive ridge resorption [14, 15]. Alveolar ridge preservation (ARP), with the application of different biomaterials, is the most common procedure aiming to control crestal bone resorption following dental extractions [16–18]. A recent systematic review confirmed that ARP results in significant reduction of vertical bone loss following dental extraction when compared to spontaneous socket healing, while its protective effect on horizontal reduction of the alveolar bone was found to be variable. Furthermore, no specific type of ARP could be demonstrated to be more effective than others in preventing alveolar crest shrinkage [19]. In a recent study, ARP performed in the posterior maxilla with a combination of allograft and collagen membrane resulted in 1.0 mm crestal height reduction and in approximately 2.5 mm loss of ridge width [20]: this volumetric contraction was lower than the one observed in extraction sites of the same area after spontaneous healing [21, 22].

Moreover, following tooth extraction in the posterior maxilla, postextractive crestal bone resorption may be associated with maxillary sinus pneumatization, which may contribute to a further decrease of the available bone volume for implant placement. The reasons for sinus pneumatization after tooth extraction are still debated and poorly understood: a possible explanation is a shift of the physiologic bone remodeling process towards a resorptive pattern, due to lack of functional forces which are normally transferred to the bone when the tooth is present [23]. This particular type of disuse atrophy occurs according to Wolff's law and is enhanced by the presence of a positive air pressure into the sinus cavity [24]. Previous human studies demonstrated a downward expansion of the maxillary sinus after dental extraction and showed that the expansion was larger if the extracted tooth was surrounded by a superiorly curved sinus floor [25, 26].

The effectiveness of ARP procedures in preventing maxillary sinus expansion has been recently evaluated in a retrospective study using bidimensional radiographs, showing significant differences in terms of postextractive pneumatization between test (ARP using bovine derived xenograft) and control group (spontaneous healing) [26].

The aim of this multicenter prospective case-control study was to evaluate, with three-dimensional analysis, the clinical effectiveness of ARP after maxillary molar extraction in reducing alveolar ridge resorption and maxillary sinus pneumatization when compared to unassisted socket healing.

## 2. Materials and Methods

### 2.1. Study Design

This multicenter prospective case-control study has been designed and conducted in accordance with the Good Clinical Practice Guidelines (GCPs) and with the recommendations of the Declaration of Helsinki for investigations with human subjects. The study protocol had been approved by the relevant Ethical Board (Comitato Etico Regione Calabria, Sezione Area Nord, n° 66/2016) and recorded in a public register of clinical trials (https://www.clinicaltrials.gov, NCT03357705).

Every patient signed an informed consent form to document the comprehension of the protocol and of the aims of this study (clinical procedures and potential risks involved). The patient had the possibility to ask questions concerning the treatment and the study protocol and was thoroughly informed about alternative therapies.

A meeting among the clinical centers was held before starting patient recruitment in order to illustrate the protocol and standardize surgical procedures. One trained oral surgeon (with more than 20 years of clinical experience) was selected in each center to perform surgical procedures, whose operative sequence was thoroughly described. The individual responsible for each clinical center received written information to standardize data collection and ensure reliable outcome reporting.

The aims of this parallel-group, multicenter prospective case-control study were the comparison of the dimensional changes of alveolar ridge and sinus floor after maxillary molar extraction with or without performing ARP.

### 2.2. Study Population

All patients who were 18 years or older and able to sign an informed consent form were considered eligible to participate in this study. Patients underwent a thorough clinical examination to evaluate the state of dentition, including periodontal and occlusal parameters, and a comprehensive treatment plan was discussed and accepted.

#### 2.2.1. Inclusion Criteria

- Indication for the extraction of a first maxillary molar presenting three separated roots;
- Presence of the adjacent teeth (second premolar and second molar);
- Presence of intact buccal and palatal bone walls (probing depth ≤3 mm);
- Absence of apical lesions with diameter >3 mm or cysts.

#### 2.2.2. Exclusion Criteria

- Acute myocardial infarction within the past 2 months;
- Uncontrolled coagulation disorders;
- Uncontrolled diabetes (HBA1c > 7.5%);
- Radiotherapy to the head/neck area within the past 24 months;
- Immunocompromised patients (HIV infection or chemotherapy within the past 5 years);
- Present or past treatment with intravenous bisphosphonates;
- Allergy to bovine collagen;
- Psychological or psychiatric disorders;
- Alcohol or drugs abuse;
- Full mouth plaque score >30% and/or full mouth bleeding score >20%;
- Necessity to perform ostectomy procedures to complete dental extraction.

### 2.3. Treatment Allocation

After a thorough discussion of the various treatment options, patients in whom an implant-supported rehabilitation was planned to replace maxillary first molar were assigned to the test group (ARP). Patients in whom a dental-supported fixed prosthesis was selected to replace the missing molar were assigned to the control group (unassisted socket healing). Patient assignment to the different groups was enclosed in identical, opaque, sealed envelopes which were opened after tooth extraction to reveal to the surgeon the treatment to be performed. Therefore, treatment allocation was concealed to the investigators in charge of enrolling and treating the patients.

### 2.4. Surgical Procedures

Patients were asked to rinse with chlorhexidine mouthwash 0.2% for 30 seconds. Under local anesthesia (Artin, Omnia, Fidenza, Italy-articain 4% with adrenaline 1:100.000), a mucoperiosteal flap was reflected and minimally traumatic extraction of the tooth was performed after separating roots with ultrasonic tips (Ninja, Acteon, Merignac, France). Roots were then mobilized by micro-elevators and individually extracted by forceps, without performing ostectomy. A careful socket debridement was performed with ultrasonic and manual instruments from the bottom of the socket up to the gingival margin, followed by an accurate removal of the sulcular epithelium. In test group (A), socket was grafted by synthetic nano-hydroxyapatite granules with 250–500 *μ*m diameter (Fisiograft Bone Granular, Ghimas, Casalecchio di Reno, Italy) and covered by haemostatic sponges with collagen of bovine origin (Hemocollagene, Septodont, Saint Maur des Fosses, France); in control group (B) socket was left to spontaneous healing without the insertion of grafting material. In both groups, flaps were mobilized with a periosteal longitudinal releasing incision and sutured with Sentineri technique [27] and single stitches reaching primary closure in both groups. Patients were prescribed with nonsteroidal antinflammatory drugs (ibuprofen 600 mg), when needed. Sutures were removed after ten days and patients entered in a follow-up protocol with periodic professional dental hygiene recalls. After six months of healing, patients were rehabilitated with dental or implant-supported prostheses according to the previously selected treatment plan.

### 2.5. Radiographic Examinations

The Ethical Board required the utilization of last generation cone beam computed tomography (CBCT) technology with maximum field of view (FOV) of 5×5 cm, in order to minimize radiation exposure. CBCTs were performed in the area of interest immediately after the end of the surgical procedure (T0) and after six months of healing (T1).

### 2.6. Quantitative Radiographic Measurements

**Figure 1:**
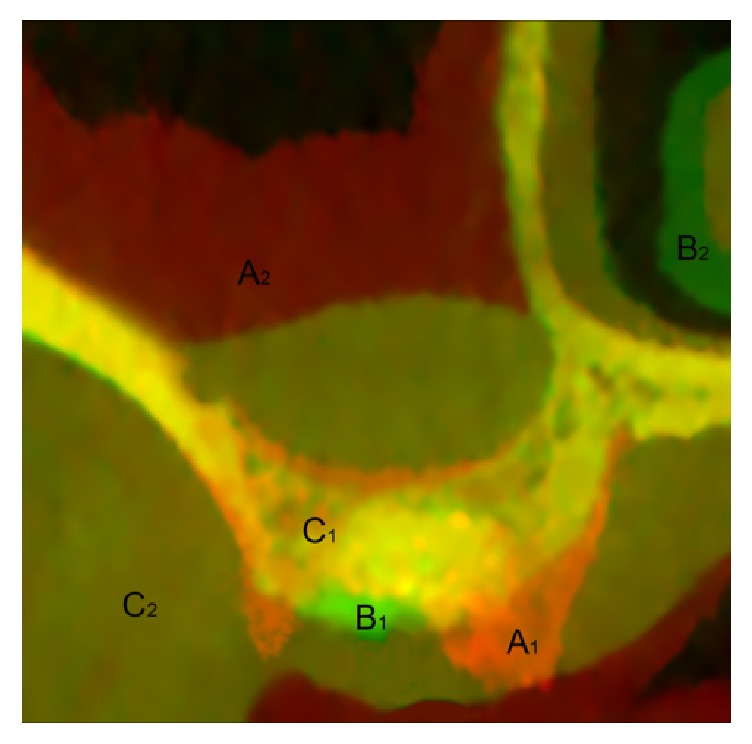
Two CBCTs of test group superimposed by Amira software, showing bone present at T0 or at T1 (A1 and B1, respectively); soft tissue present at T0 or at T1 (A2 and B2, respectively), and bone and soft tissues present both at T0 and T1 (C1 and C2, respectively). Schneiderian membrane hypertrophy appears considerably reduced during the healing period.

T0 and T1 CBCTs of each patient were uploaded on an advanced 3-D image processing and quantification software (Amira, Thermo Fisher Scientific, Waltham, USA). Fiji open-source software [28] was used to perform linear measurements after superimposing CBCTs as previously described by Ryckman et al. [29] (Figure 1). In detail, CBCTs were superimposed by using dental and osseous structures unaffected by the surgery as landmarks (adjacent teeth, lateral and medial maxillary sinus walls, and palatine process): after selecting these regions, the automatic affine registration tool of the software was used for stepwise superimposition. All measurements were taken by a single-blinded calibrated examiner (DP) on a 30-inch led-backlit colour diagnostic display and each measurement was repeated three times at three different time points as proposed by Gomez-Roman and Launer [30]. Examiner calibration was performed by assessing five CBCTs, with another author (FB) who served as reference examiner. Intraexaminer and interexaminer concordances were 95.5% and 91.7%, respectively, for linear measurements within ±0.1 mm.

**Figure 2:**
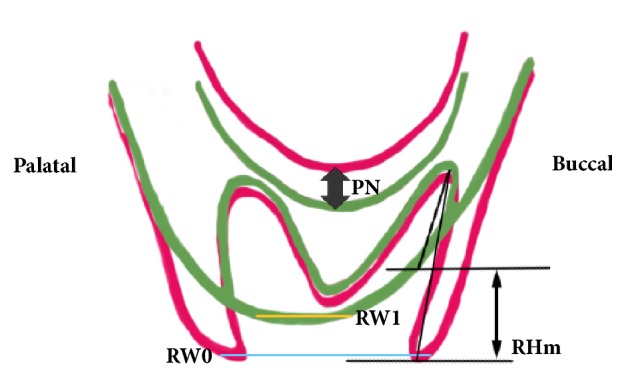
Diagram summarizing main reference points taken for measurements.** PN**: sinus expansion between T0 and T1 (mm).** RHm**: vertical reduction of the alveolar bone measured (mm) in correspondence of the mesial socket, following the root axis and representing the linear difference between the most coronal position of the ridge at T0 and T1 and the root apex at T0.** RW0**: ridge width in the most coronal part of the crest at T0.** RW1**: ridge width in the most coronal part of the crest at T1.

In detail, entity of maxillary sinus expansion between T0 and T1 was measured (mm) in correspondence to the apex of the three sockets, following the root axis (PNp, PNd, and PNm) and in the center of the crest (PN). Vertical reduction of the alveolar bone was measured (mm) in correspondence to each of the three sockets, following the root axis, and represented the linear differences between the most coronal position of the ridge at T0 and T1 and the root apex at T0 (RHp, RHd, RHm). Furthermore, alveolar bone height (RH) was measured in the center of the crest (mm), from the most coronal part of the ridge to the sinus floor, both at T0 and at T1. Finally, horizontal reduction of the ridge (RW) was the difference (mm) between bone width in the most coronal part of the crest at T0 (RW0) and T1 (RW1).  Figure 2 summarized the reference points taken for measurements.

### 2.7. Outcomes

This study evaluated the following outcome measures:

*Primary Outcomes*

- Maxillary sinus pneumatization (mm): mean of 4 measurements of sinus floor expansion (PNm, PNd, PNp, and PN) from T0 to T1;
- Alveolar bone dimensional changes (mm): vertical (mean among RHm, RHd, and RHp; RH) and horizontal reduction (RW) of the alveolar bone from T0 to T1.

*Secondary Outcomes*

- Biological complications: any complication defined as an unexpected deviation from the normal treatment outcome (e.g., alveolitis, postoperative infections).

### 2.8. Sample Size and Statistical Power

The calculation was performed with a specific software (DSS Research, Fort Worth, USA) to detect a significant difference between the groups in terms of maxillary sinus pneumatization, based on the outcomes of previous research (0.5 mm with an expected standard deviation of 0.3 mm) [26]. A sample of 14 patients (7 test and 7 control cases) was needed to reach 80% of statistical power with *α* set at 0.05. Each clinical center treated 10 patients for a total of 30 (15 test, 15 control) to compensate eventual drop-outs occurring during the follow-up period.

### 2.9. Statistical Analysis

Statistical analysis was performed by means of OriginLab software (OriginLab Corporation, Northampton, USA). Data for descriptive statistics were expressed as mean±SD. All data (with one exception) satisfied both the normality (Kolmogorov–Smirnov test) and the equality of variance (Levene test) assumptions and were analyzed with independent samples T-test. Only RHm of both test and control groups after six months of healing did not satisfy normality and equality of variance and were analyzed by Mann–Whitney test. Fisher exact test was used to evaluate age and gender distribution between the groups. Statistical significance was preset at *α*=0.05.

## 3. Results

Thirty consecutive patients (14 males and 16 females, age range between 27 and 75, mean 52.7±7.4, 3 smokers, and 27 nonsmokers) were enrolled and treated with maxillary first molar extraction followed (n=15) or not followed (n=15) by ARP procedures. Complete demographic characteristics of the sample are listed in Table 1: in particular, test and control groups were balanced for age and gender.

**Figure 3:**
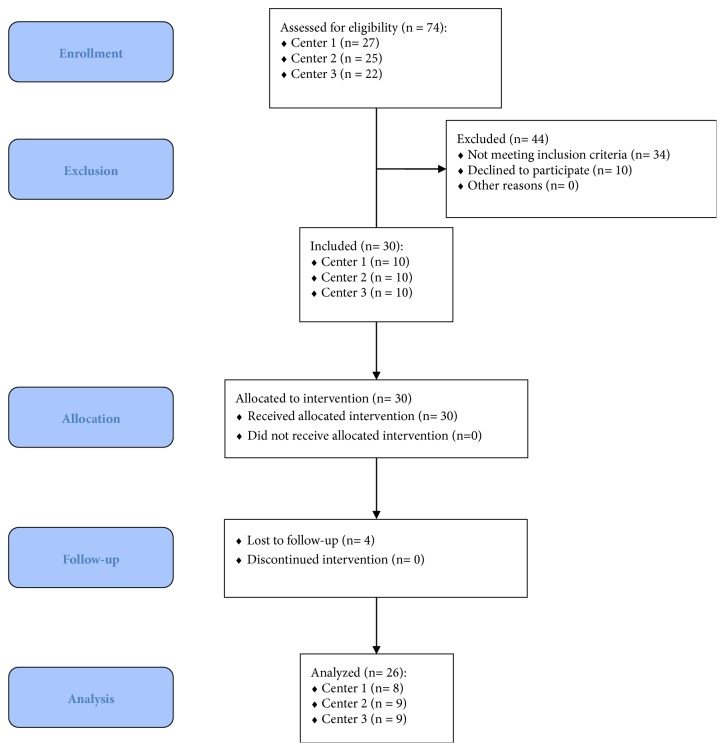
Selection process of patients participating in this study.

Surgeries were performed by three experienced operators [TL (Center 1) n=10; FaB (Center 2) n=10; CS (Center 3) n=10] between November 2016 and June 2017. Four patients (test n=2; control n=2) dropped out at 6-month follow-up (one patient moved abroad, three patients did not come to the control visit within the six month after tooth extraction). Twenty-six patients (test n=13; control n=13) were included in the final analysis. A flowchart diagram summarizing patient selection process was presented in Figure 3.

Six months after dental extraction, sinus floor expansion (mean of PNm, PNd, PNp, and PN) was 0.69±0.48 mm in the test group and 1.04±0.67 mm in the control group (p=0.15). Complete results are reported in Table 2.

Vertical resorption of the alveolar bone (mean of RHm, RHd, and RHp) after six months of healing was 1.62±0.49 mm in the test group and 2.01±0.84 mm in the control group (p=0.08). Complete data are listed in Table 3.

Available bone height (RH) at T0 was 8.34±3.25 mm and 6.40±1.64 mm in test and control group, respectively (p=0.07). At T1, RH was 8.01±3.49 mm and 5.34±2.11 mm in test and control group, respectively (p=0.03). Mean vertical bone loss after six months of healing was 0.33±1.94 mm in test group and 1.06±0.93 mm in control group (p=0.23).

Alveolar bone width (RW) at T0 was 11.27±1.71 mm and 12.06±2.46 mm in test and control group, respectively (p=0.35). At T1, RW was 8.54±1.26 mm and 8.43±2.26 mm in test and control group, respectively (p=0.89). Mean horizontal resorption of crestal bone after six months of healing was 2.73±1.68 mm in test group and 3.63±2.24 mm in control group (p=0.24).

No biological complications were recorded during the healing phase. After six months, thirteen patients of the test group underwent dental implant insertion: twelve patients underwent standard implant placement (92.3%); sinus floor elevation was performed in one patient in order to allow implant insertion (7.7%).

## 4. Discussion

To the best of our knowledge, this paper represents the first prospective case-control study evaluating, with three-dimensional analysis, the clinical effectiveness of ARP in reducing alveolar ridge resorption and maxillary sinus pneumatization after first molar extraction when compared to unassisted socket healing. Previous studies on this topic consisted in retrospective studies based on bidimensional radiographs (orthopantomographies) [23, 25, 26], which do not provide images in bucco-palatal cross section and could be hampered by image distortion [31]. In the present prospective study, measurements were performed by using a semiautomatic method for the comparison of three-dimensional images basing on CBCT superimposition, introduced for the evaluation of bone changes after ARP by Clozza et al. [32]. The accuracy of this procedure is directly dependent on the precision of the superimposition of baseline and final CBCTs: however, possible errors (related to scan acquisition or to incorrect evaluation of the stable regions) are usually negligible, and maxillary regional CBCT superimposition is currently considered as an accurate and reliable method [33].

In the present study, mean extent of sinus pneumatization measured in spontaneously healed sockets six months after tooth extraction was 1.04±0.67 mm, in substantial agreement with previous works by Sharan and Madjar (1.83±2.46 mm) [25] and Levi et al. (1.30±0.27 mm) [26]. Conversely, a smaller downward expansion of the sinus floor, even if not significant, was observed in sites treated with ARP (0.69±0.48 mm), in accordance with the general trend reported by Levi et al. (0.30±0.10 mm) [26].

Hence, these findings suggested that ARP performed after maxillary molar extraction could be an effective technique in reducing maxillary sinus floor pneumatization: mean difference in sinus expansion between test and control group was 0.35 mm after six months of healing. Also this outcome is in accordance with the study of Levi et al. [26], who reported a protective action of ARP on the extent of postextractive sinus pneumatization, even if with a significantly higher mean difference between test and control groups (1 mm).

**Figure 4:**
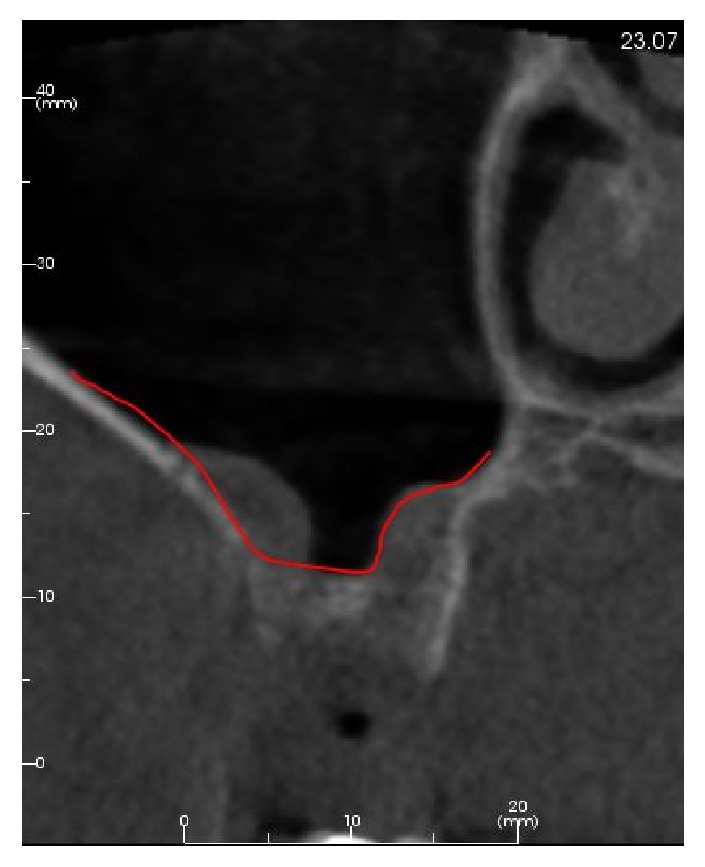
An example of a particular anatomical situation in which the lack of three-dimensional evaluation could lead to misleading interpretations of available bone height.

Postextractive bone remodeling is a widely studied phenomenon regulated by a series of biological events mainly involving bundle bone resorption and resulting in reduced height and width of the residual alveolar ridge [2]. Many clinical trials and systematic reviews with meta-analysis demonstrated that, in general, both horizontal and vertical resorption are more pronounced on the buccal side [34, 35] and that the horizontal reduction (weighted mean 3.87 mm) is greater than the loss in height (weighted mean 1.53 mm) [36]. Alveolar socket grafting was first proposed in the mid-1970s in the attempt to reduce the need of subsequent ridge augmentation procedures prior to implant placement [37, 38]. Many ARP techniques have been proposed lately, improving and extensively testing with various different surgical approaches and biomaterials. Recent systematic reviews with meta-analysis confirmed the effectiveness of ARP in reducing postextractive horizontal and vertical alveolar ridge resorption when compared to spontaneous socket healing, even if the superiority of a specific biomaterial or surgical approach has not been demonstrated yet [17–19, 39–42]. In these studies, the reported clinical magnitude of the effect ranged from 1.4 to 2.19 in terms of buccolingual width and from 1.02 to 2.6 mm in terms of crestal height: the outcomes of the present study (difference between ARP and untreated socket of 0.9 mm in terms of horizontal reduction and 0.39 mm in terms of vertical bone loss) are in line with this trend even if they result in lower values. This occurrence could be possibly explained by the fact that the present study is one of the very few using three-dimensional image processing and measurement tools, which have an higher accuracy and possibility of deeper evaluations than the commonly used bidimensional image analysis systems [43, 44]. Furthermore, the lack of a three-dimensional evaluation could lead to misleading interpretations of particular anatomical conditions, both for clinical and for research purposes (Figure 4).

On this basis, the use of ARP procedures after the extraction of a maxillary molar could be regarded as a preventive treatment, particularly in cases where an implant-supported restoration is planned. Maxillary molars (particularly first molar) are the sites in which the combined action of alveolar bone remodeling and maxillary sinus pneumatization leads to the greater amount of vertical bone loss after tooth extraction of the entire upper arch [4, 23]. Hence, a preservation of vertical and horizontal alveolar ridge dimensions could often be clinically significant, allowing a standard implant placement without additional regenerative procedures. Sinus floor elevation (both lateral and transcrestal) and GBR techniques are advanced procedures requiring specific operator skills and are always associated with higher morbidity, increasing costs, prolonged time of therapy, and potential intra- and postoperative complications [12, 45–48]. In our test group, mean available crestal height (RH) was 8.34 mm at baseline and 8.01 mm six months after molar extraction, presenting only a 4% reduction: 12 patients out of 13 underwent implant insertion without the need of additional bone augmentation procedures. This finding is in accordance with the results of a randomized trial by Rasperini et al. [49], confirming that ARP performed in the posterior maxilla increases the possibility of inserting implants without the need for contextual sinus augmentation procedures.

Some limitations should be considered when interpreting the outcomes of this study. The first to consider is the strict local inclusion criteria (three separated roots; presence of the adjacent teeth; presence of intact buccal and palatal bone walls): ARP effectiveness should be tested also in different conditions. Secondly, the use of CBCT as three-dimensional data source, even if more reliable than bidimensional radiographs, may be subjected to lack of standardization among different equipment. Third, no histologic evaluations have been performed in the present study: even if the use of synthetic grafts in ARP is supported by recent randomized controlled trials with human histologies [50, 51], further research is necessary to confirm the effectiveness of this class of biomaterials in this particular application.

Finally, randomized clinical trials on larger samples are recommended, in order to prevent potential bias in patient allocation to the different treatment groups and to reduce data dispersion.

## 5. Conclusion

Within the limitations of this study, it could be suggested that ARP performed after maxillary molar extraction may reduce the entity of sinus pneumatization and alveolar bone resorption, compared to unassisted socket healing. The possibility of an effective preservation of vertical and horizontal crestal dimensions could decrease the necessity of advanced regenerative procedures prior to dental implant placement: further studies are necessary to confirm these findings and to better define the clinical magnitude of the effect.

## Figures and Tables

**Table 1:** Demographic characteristics.

**Gender**	**Age**			**Sample size**		
	**Test**	**Control**	**Diff.**	**Test**	**Control**	**Diff.**
**Males**	53.4 ± 7.3	51.9 ± 6.5	0.342;** NS**	7	7	1;** NS**
**Females**	56.1 ± 4.2	55.4 ± 5.8		8	8	

Age is presented as mean±standard deviation. **Diff.**, significance of the difference between the groups. **NS**, no significant difference.

**Table 2:** Entity of sinus pneumatization six months after dental extraction.

	**Control**	**Test**	**Difference**	**Diff.**
	**(mm)**	**(mm)**	**(mm)**	
**PNm**	0.82 ± 0.38	0.59 ± 0.43	0.23	0.15; **NS**

**PNd**	0.79 ± 0.71	0.55 ± 0.51	0.24	0.33; **NS**

**PNp**	1.08 ± 1.03	0.72 ± 0.70	0.36	0.3; **NS**

**PN**	1.46 ± 0.93	0.92 ± 0.63	0.54	0.1; **NS**

**Total**	1.04±0.67	0.69±0.48	0.35	0.15; **NS**

Measures are presented as mean±standard deviation. **PNm**, **PNd**, **PNp**, **PN**, sinus expansion between T0 and T1 measured following the axis of the mesial **(PNm)**, distal **(PNd)**, palatal **(PNp)** roots and in the center of the crest **(PN)**. **Diff.** significance of the difference between the groups. **NS**, no significant difference.

**Table 3:** Vertical resorption of the alveolar bone six months after dental extraction.

	**Control**	**Test**	**Difference**	**Diff.**
	**(mm)**	**(mm)**	**(mm)**	
**RHm**	2.52 ± 1.52	1.45 ± 0.64	1.07	0.20; **NS**

**RHd**	1.75 ± 1.24	1.74 ± 0.57	0.01	0.97; **NS**

**RHp**	2.31 ± 1.50	1.67 ± 1.02	0.64	0.14; **NS**

**Total**	2.01 ± 0.84	1.62 ± 0.49	0.39	0.08; **NS**

Measures are presented as mean±standard deviation. **RHm**, **RHd**, **RHp**, vertical reduction of the alveolar bone expressed as the difference between the most coronal positions of the ridge at T0 and T1 and the root apex at T0, measured following the axis of the mesial **(RHm)**, distal **(RHd)** and palatal **(RHp)** roots. **Diff.** significance of the difference between the groups. **NS**, no significant difference.

## Data Availability

The datasets generated and analyzed during the current study are available from the corresponding author upon reasonable request.
